# Cytoplasmic physicochemical factors drive malignant transformation by adapting bioenergetic settings

**DOI:** 10.1016/j.bbrep.2025.102079

**Published:** 2025-06-07

**Authors:** Tattym E. Shaiken

**Affiliations:** Department of Medicine, Baylor College of Medicine, Houston, TX, 77030, United States; PeriNuc Labs, University of Houston Technology Bridge, Houston, TX, 77023, United States

## Abstract

While significant insights have been gained by a study of cancer genetics, the roles of the cytoplasm in regulating chemical processes during the transformation to malignancy are often less appreciated. The cytoplasm functions as a two-phase system consisting of an elastic solid phase (the cytomatrix) and a viscous liquid phase (the cytosol). This finding has led to the development of a tumor progression model based on a two-phase system that connects genetic alterations with the physicochemical processes necessary for sustaining and facilitating malignant growth. Here, we show that the energy required for tumor growth is, in part, required for the cytomatrix activity, which accelerates chemical reactions. The ability to regulate cytomatrix motor proteins provides a mechanism to control whether a genetic mutation is able to induce the energy needed for cancer to develop and offers innovative strategies for cancer treatment.

## Highlights

• Cytoplasmic fluctuations serve to overcome viscosity and diffusion limitations.
• The cytoplasm consists of two physical phases: solid cytomatrix and fluid cytosol.
• Non-muscle actomyosin generates forces that drive the cytomatrix dynamics.
• Cytomatrix drives cytosolic flow, delivering substrates to immobilized enzymes.
• Increased activity of the cytomatrix contributes to the Warburg effect in cancers.

## Graphical abstract

**Figure:**
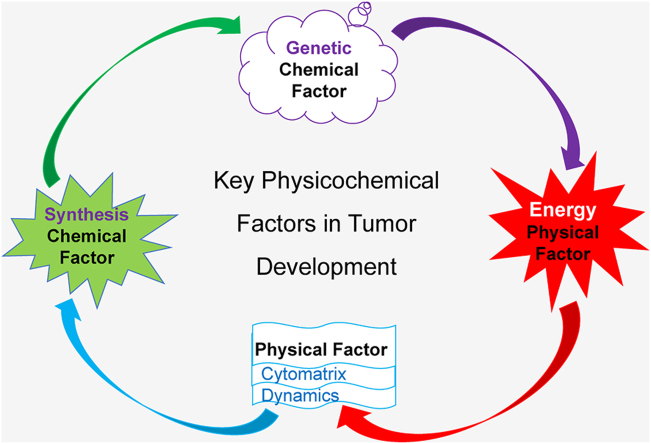

## 1 Introduction

Gene mutations provide a mechanism for adaptation to environmental changes and are a driving force for species evolution. Unfortunately, mutations may also trigger cancer. Recent studies show that factors besides genetic mutation contribute to malignant transformation [1]. Insights into carcinogenesis can be found in cancer characteristics such as nucleolar hypertrophy, a histologic characteristic of tumors first observed a century ago. Nucleolar hypertrophy describes the enlargement of the nucleolus and indicates increased protein translation, cell growth, and proliferation [2]. Another early finding that was characteristic of malignant tumors (known as the Warburg effect) is that they frequently overproduce lactic acid through aerobic glycolysis. Understanding the link between cancer traits and genetic abnormalities can deepen our insights into malignant transformation and clarify cellular physicochemical processes.

The chemical reactions within cells typically occur in a gel-like solid [3], which is a viscous and very crowded environment where free diffusion is restricted. Furthermore, molecular diversity results in low concentrations of individual components despite crowded conditions, which suggests that the law of mass action may not be applicable [4]. Although theoretically, chemical reactions should occur extremely slowly within the cytoplasm due to spatial constraints, calculations indicate that millions of chemical reactions take place in cells every second. As a result, textbooks often describe the mechanisms of these chemical reactions as if the cell were simply in an aqueous solution, relying on the concept of free diffusion [5]. The dichotomy between the chemical processes and the physical condition of the cell cytoplasm, where the primary biochemical processes occur, has been unexplained. A better understanding of this paradox should clarify how cell chemistry is organized and how it contributes to cancer.

To address this paradox, we considered the cell cytoplasm as having two physical phases: an elastic solid phase, which we named the cytomatrix, and the viscous fluid phase, the cytosol [6]. Our data indicate that the cytomatrix, besides containing cytoskeletal proteins such as actin, myosin, and tubulin, is rich in enzymes, suggesting that catalytic complexes are fixed within the solid phase. Immobilized enzymes can operate without spatial constraints, thus enabling various chemical processes to occur simultaneously without interference. Efficient chemical reactions also depend on movements of the cytosolic liquid phase, which can be facilitated by motor proteins within the solid phase. The high activity of these motor proteins requires sufficient energy to enhance the rate of chemical reactions, especially in cancer cells. Here, we propose a physicochemical model of malignant transformation based on the ribosome footprint of the cytomatrix and cytosol, and mass spectrometry data combined with the contribution of actin and myosin in lactic acid production.

## 2 Materials and methods

### 2.1 Cell line, cell culture, cell fractionation, and chemicals

Colorectal carcinoma HCT-15 (CCL-225) cells, purchased from ATCC (Manassas, VA 20110, USA), were grown in an incubator under humidified conditions at 37 °C under 5 % CO_2_ in RPMI-1640 supplemented with 10 % (v/v) FBS and 1 % (v/v) penicillin and streptomycin. For the l-lactic acid assay, the medium containing actin and myosin inhibitors was used. NAD (cat #N7004), L-LDH (cat #L125). M-PMS (cat #M8640), INT (cat #I8377), CK 666 (cat # SML0006), Wiskostatin (cat # 681525), Narciclasine (cat # SML2805), SMIFH2 (cat #S4826), ML-7(cat # 475880), (±) Blebbistatin (cat # 203390), and pp242 (cat # 475988) were purchased from Sigma-Aldrich (St.Louis, MO, USA). The method of cell fractionation into the cytosol and cytomatrix as described previously [6]. Briefly, cells were lysed using Buffer A (40 mM Hepes pH 7.4, 120 mM KCl, 0.5 % Glycerol, and 0.5 % NP-40 with protease inhibitors). Lysates were centrifuged at 500×*g* for 5 min at 4 °C, and the supernatants were collected as the cytosol. The pellet was resuspended by gently pipetting in Buffer B (10 mM Tris-HCl pH 7.5, 1.5 mM KCl, 0.5 % Triton X-100, 0.5 % sodium deoxycholate, 2.5 mM MgCl2, 0.2 M LiCl, and protease inhibitors). The resuspension of the pellet by cold rotation continued for 45 min to complete solubilization. The cytomatrix was isolated by centrifuging at 2000×*g* for 5 min. The supernatants were collected as the cytomatrix.

### 2.2 LC-MS/MS analysis

Two biological replicates were used for LC-MS/MS analysis. The cytomatrix fractions were concentrated and digested on an S-Trap™ column per the manufacturer's protocol. Offline high pH STAGE peptide fractionation for 50 μg peptides was carried out. LC-MS/MS analysis was carried out using a nano-LC 1200 system coupled to Orbitrap Lumos ETD mass spectrometer. The peptides identified from the Mascot result file were validated with 5 % false discovery rate. The median normalized iBAQ values were used for data analysis. The differentially expressed proteins were calculated using the moderated *t*-test to calculate p values and log2 fold changes in the R package limma. The FDR corrected p value was calculated using the Benjamini-Hochberg procedure. Gene Set Enrichment Analysis was performed using the canonical pathway gene sets derived from the KEGG and Reactome pathway databases.

### 2.3 Ribo-seq

The cytosol and cytomatrix from control and treated cells were obtained as previously described and digested with micrococcal nuclease (MNase, Sigma-Aldrich, St Louis, MO) at 37 °C for 30 min to obtain ribosome footprints. The reaction was stopped by the addition of 1.5 vol of 4 M guanidine thiocyanate. Footprints were extracted by adding TRIzol and isolated by precipitating with GlycoBlue overnight [7]. The footprints were treated with polynucleotide kinase at 37 °C for 30 min to reverse the phosphate position. We separated 17–37 nt long footprints from the gel under UV light. We froze gel slices at −80 °C and then crushed them to extract RNA.

### 2.4 Library preparation

We used 10 ng of size-selected ribosome-protected RNA (17–37 nt) as the starting material for the QIAseq miRNA Library Prep Kit (cat #331502). RNA fragments were ligated to adapters at the 3′ and 5′ ends, reverse transcribed, and amplified. The resulting libraries were size selected for 185–191 bp fragments. The libraries were quantitated by qPCR using the Applied Biosystems ViiA7 Quantitative PCR instrument and a KAPA Library Quant Kit (p/n KK4824). All samples were pooled equimolarly and sequenced on a NextSeq 500 High Output v2.5 flowcell using the Illumina NextSeq 500 sequencing instrument with a single-read configuration (75 bp). An average of 42 million reads per sample was sequenced. FastQ file generation was executed using Illumina's cloud-based informatics platform, BaseSpace Sequencing Hub.

### 2.5 Bulk ribo-seq analysis

Two biological replicates of ribosomal footprint and mRNA libraries were generated and subjected to deep sequencing to obtain the Ribo-seq data sets as described [6]. ORA was performed to detect enriched gene sets corresponding to pathways and biological processes based on differential gene expression. Using the KEGG and the Molecular Signature Database methodology, we assessed enrichment with a hypergeometric test. Significance was achieved at an FDR-adjusted p value < 0.05.

### 2.6 l-lactate level measurement

HCT-15 cells were seeded in 24-well plates at a density of 0.1 x 10^6^ cells/well. After 48 h, the growth medium was replaced with the medium containing different concentrations of CK 666, Wiskostatin, ML 7, Blebbstatin, SMIFH2 (5, 10, 15 μM), Narciclasine (0.62, 1.25, 2.5 μM) or untreated control and were incubated for 24 h, at 37 °C in 5 % CO_2_. For the time-course experiments, 50 μl of supernatant from each well was transferred to 96-well plates. The colorimetric l-lactate assay was conducted following the method described by Schmiedeknecht et al. (2022) [8]. Four replicates of supernatant were each mixed with 50 μl of L-lactic assay reaction mix. The mixture was then incubated for 2 h at room temperature in the dark. The reaction was stopped by adding 50 μL of 1 mM acetic acid, and the absorbance was measured using a microplate reader at 490 nm with a reference wavelength of 650 nm.

### 2.7 Statistical analysis

Data are presented as the mean ± SD. One-way ANOVA was used to detect significant differences between the means of more than two independent groups using Prism 9 (GraphPad). We independently performed t-tests to confirm statistically significant differences between two groups. A p value of <0.05 was considered as significant.

## 3 Results

### 3.1 Ribosome footprint analysis

The separation of the cytosol and cytomatrix from the nucleus has been previously described in four cell types, including normal primary MEF cells, highlighting differences between the cytosol and cytomatrix [6,9]. To address the contribution of cytomatrix activity in tumor development, the ribosome footprint and mass-spectrometry data were analyzed. Ribosome footprint analysis, known as Ribo-seq, was utilized to identify differentially expressed genes. In this study, the KEGG collection of databases was employed to differentiate pathways of the cytomatrix and cytosol. The over-representation analysis of the cytomatrix relative to the cytosol was conducted for the cytomatrix dataset.

**Fig. 1:**
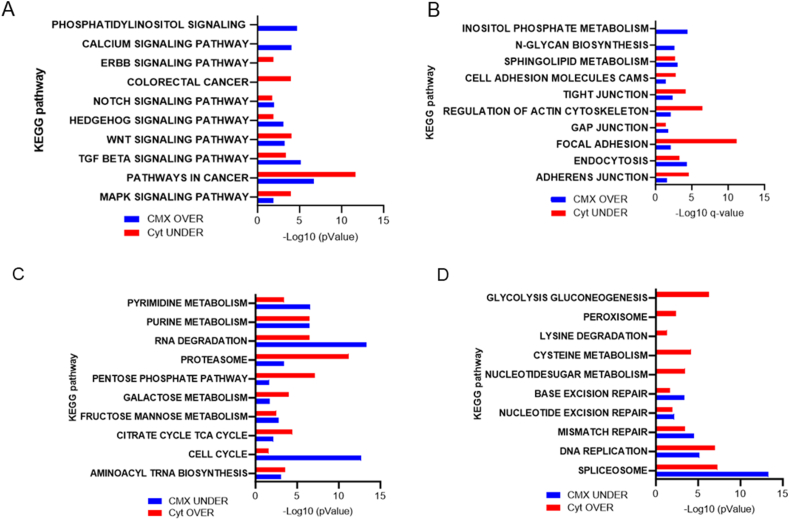
**KEGG-based analysis of ribosome footprints of the HCT-15 cells**. (A, B) Overrepresented pathways in the cytomatrix and underrepresented pathways in the cytosol. (C, D) Overrepresented pathways in the cytosol and underrepresented pathways in the cytomatrix. Overrepresentation analysis (ORA) significance was determined as the –Log 10 q-value. Significance was achieved at an FDR-adjusted p-value <0.05. CMX – cytomatrix, Cyt – cytosol, OVER – overrepresented and UNDER – underrepresented pathways.

Ribo-seq revealed a distinct functional distribution of polysomes between the solid and liquid phases (Supplementary Table S1 and S2). The cytomatrix was enriched with receptors, signaling pathways, and components of the extracellular matrix (Fig. 1A and B). The signaling pathways essential for normal development, whose disruption may result in cancer development (Fig. 1A), were enriched yet underrepresented in the cytosol. Additionally, cell-to-cell communication junctions, which are crucial for maintaining the structural integrity of tissues and regulating actin filaments, were overrepresented.

In contrast, carbohydrate, protein, and nucleic acid metabolism and degradation pathways were overrepresented within the cytosol and underrepresented in the cytomatrix (Fig. 1C and D). This contrast between the cytomatrix and cytosol pathways highlights the unique protein synthesis capacities of solid and liquid phases, underscoring the cytomatrix's role in protein synthesis required for cancer development.

### 3.2 Mass-spectrometry profile of the cytomatrix proteome

**Fig. 2:**
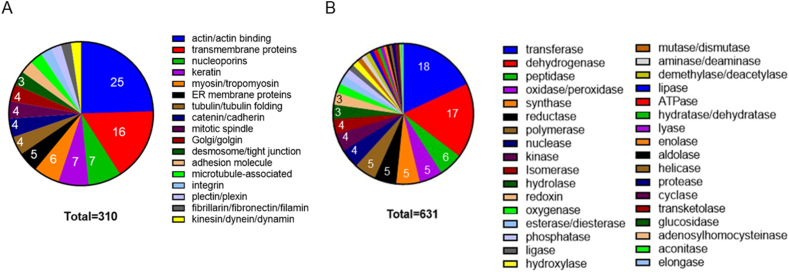
**Mass-spectrometry analysis of the cytomatrix proteome.** Pie charts illustrate (A) the structural proteome and (B) the enzyme spectrum of the HCT-15 cell cytomatrix. Proteins in the group were selected according to the intensity-based absolute quantification (iBAQ) score. The percentage for each group was calculated based on the total number of selected proteomes in each collection, which is shown within the slices.

Mass spectrometry-based proteomics was performed to analyze the cytomatrix proteome (Supplementary Table S3, Structure and Enzymes). The proteins that form the structural basis of the cytomatrix, including actomyosin and other actin-binding proteins, were the most abundant and diverse among structural proteins (Supplementary Table S3 and S4). They made up about a quarter of all identified structural proteins (Fig. 2A). Four types of actins, three isoforms of actinins, five types of tropomyosins, twelve classes of myosins, and a total of one hundred fifty actin-interacting proteins were identified within the cytomatrix (Supplementary Table S5). Monomeric globular actin (G-actin) is an ATPase that can self-assemble into filamentous actin (F-actin) in the presence of ATP. Actin-binding proteins play a crucial role in regulating the rate of actin polymerization and affecting the length of actin filaments [10]. Examples of proteins that affect the speed of actin polymerization and the length of actin filaments include cofilin, formin homology (FH) proteins, and the actin-related protein complex (Arp2/3 complex). The motor protein myosin and its binding proteins were also abundant in the cytomatrix proteome. Non-muscle cells express multiple myosin isoforms with specialized cellular roles and minimal overlapping functions [11]. Our finding suggests that actomyosin motor proteins may be the primary mechanical force in the cytomatrix. This conclusion is supported by a direct experiment in which intracellular fluctuations were suppressed by blebbistatin, which inhibits actomyosin contraction [12]. The cytomatrix was also enriched in proteins from the Golgi apparatus, endoplasmic reticulum, nuclear pore complex, and nuclear envelope and plasma membranes. This indicates that the solid phase consists not only of cytoskeletal proteins but also of the protein carcasses of organelles, with nuclear and plasma membranes forming part of a solid structure (Fig. 2A).

The diverse range of enzymes (Supplementary Table S3 and S4) in the cytomatrix suggests that metabolic reactions, which could potentially inhibit one another, may occur simultaneously, likely due to their immobilization on the cytomatrix and spatial segregation (Fig. 2B). Immobilization and segregation help overcome spatial barriers to chemical reactions. Enzymes attached to the cytomatrix need the flow of cytosol for their reactions. The motor proteins of the cytomatrix can facilitate the movement of substrates to catalytic complexes [6].

### 3.3 Effects of actomyosin modulators on l-lactic acid generation

Anaerobic glycolysis is the initial step in aerobic respiration, produces ATP significantly faster than oxidative phosphorylation [13]. Cells that use energy resources more rapidly benefit from a higher rate of ATP production, and glycolysis is an adaptable energy production process that supplies additional energy for cells when necessary [14]. Malignant cells may require more energy than can be produced by the limited number of mitochondria through the process known as aerobic glycolysis or the Warburg effect. The Warburg effect is no longer thought to be essential for obtaining biomass precursors, highlighting its role in energy generation, as oxygen consumption increases in Warburg-null cells [15]. However, the intracellular process that benefits from energy production through aerobic glycolysis remains unclear.

**Fig. 3:**
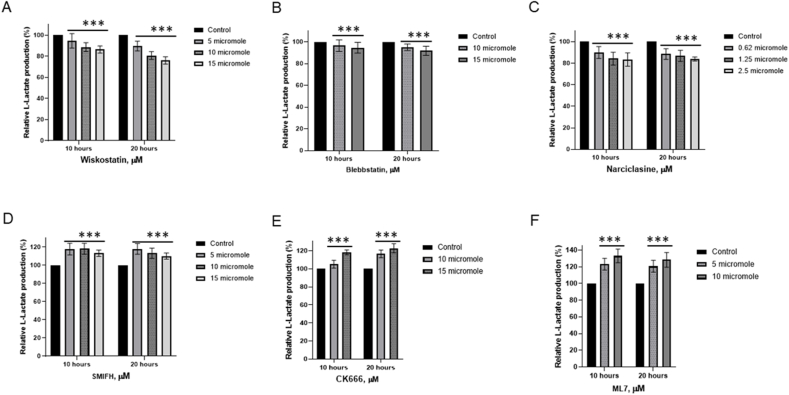
**l****-Lactate analysis of HCT-15 cell culture using LDH assay.** HCT-15 cells were seeded in 24-well plates. After 48 h, growth medium was replaced with medium containing the indicated concentrations of compounds. 50 μL supernatant aliquots were transferred to 96-well plates at 10 and 20 h. Four replicates of supernatant in duplicate were each mixed with 50 μL of the LDH assay reaction mix. The mixture was then incubated for 2 h at room temperature in the dark, stopped by adding 50 μL of acetic acid. The absorbance was measured at 490 nm. The data represent the mean ± SD of four replicates. ∗∗∗p < 0.001 compared to untreated.

We hypothesized that ATP generated by glycolysis could fulfill the energy requirements for the micromechanics of the cytomatrix for actin, as another motor protein, tubulin, relies on GTP [16,17]. To test this hypothesis, we examined actin and myosin modulators for their impact on lactate production in the HCT-15 colon cancer cell lines (Fig. 3).

Wiskostatin, Blebbistatin, and Narciclastin exhibited varying degrees of suppression in lactic acid production (Fig. 3A–C). Wiskostatin selectively suppresses WASP (Wiskott-Aldrich syndrome protein)-mediated actin polymerization [18]. Narciclasine impairs the organization of the actin cytoskeleton by targeting RhoA GTPases [19]. Blebbistatin, an ATPase inhibitor selective for myosin II [20].

On the contrary, SMIFH2 and CK 666 actin modulators, increased lactate generation in colon cancer cells (Fig. 3D–E). SMIFH2 inhibits actin polymerization by binding formins [21] and suppresses myosin ATPase activity [22]. CK 666 disrupts actin polymerization by locking the Arp2/3 complex in an inactive conformation [23] and protects against ferroptosis, the iron-dependent form of programmed cell death [24]. Myosin Light Chain Kinase (MLCK) Inhibitor, ML-7, also increased l-lactate generation (Fig. 3F). MLCK regulates contractions through myosin activation [25]. ML-7 has been shown to protect cardiac function by increasing energy production [26].

Modulators of actomyosin activity can both increase and decrease lactate production. Compounds that increased lactic acid generation were involved in modulating several additional pathways, which may have a protective role, supporting our previous finding that inhibiting different signaling pathways can lead to an increase in metabolic activity as a compensatory mechanism [6]. The modulation of lactic acid production by regulators of actomyosin activity supports the concept that, in tumors, intracellular motion relies on aerobic glycolysis, similar to how skeletal muscle uses glycolysis during intense exercise.

## 4 Discussion

We linked the non-muscular cells' actomyosin with aerobic glycolysis, known as the Warburg effect. Intracellular chemical processes are influenced by cytoplasmic fluctuations driven by the dynamics of the cytomatrix [6]. In this context, the cytoplasm's viscosity is an essential factor for regulating cellular chemical processes in coordination with the motor proteins to achieve a physiological rate of metabolism. The actin superfamily of proteins converts chemical energy from ATP into mechanical energy. Actin filaments continuously polymerize and depolymerize at the astonishing rate of up to several hundred subunits per second in cells [27]. Proteins linked to filaments can rearrange in milliseconds, and molecular motors are able to experience cyclical conformational changes at rates exceeding 100 Hz [28]. The fact that actin polymerization and depolymerization occur so rapidly leads to the question of how they can also function as parts of the cytoskeleton, leading to the question that, given the large number of binding and regulating proteins involved, whether there is another, as yet unknown function that has not been considered?

Extracellular signals regulate actin and actin-binding protein dynamics, and time-lapse images have recently shown a role for the adherence junction in the actin dynamics [27]. Molecular motors in cells typically produce highly directed motion [12], with the sum of this motor protein activity being visualized as random fluctuations of the cytoplasm. Many extracellular and intracellular actin regulators control the rate of chemical processes that deliver substrates to immobilized complexes, generating a tunneling effect for a brief period to ensure that the chemical reaction is completed.

A novel perspective on malignant transformation is related to the link of actin dynamics in non-muscle cells to lactic acid production (the Warburg effect) and cellular chemistry. The rate of enzymatic processes is regulated by the mechanical activity of actin, with the required energy provided by ATP to overcome the diffusion limitations of the viscous medium. This process supplies substrates to catalytic complexes. Thus, one main difference between healthy and malignant cells may lie in the rate of chemical reactions. Mitochondrial respiration and regulated cytomatrix actin mechanics and controlled biosynthesis [29] maintain organized cell division. The cytomatrix micromechanics working through actin dynamics is critical in supporting the steady-state kinetics of catalytic processes within the cytoplasm. Although many enzymes exhibit a relatively high catalytic turnover number, cellular processes are sustained by the gradual progression of chemical processes. The physical properties of the cytoplasm, such as viscosity, provide an overall moderate physiological rate of metabolism. Additionally, enzymatic feedback regulation contributes to slowing the generation of the final product when necessary.

In contrast, cancer cells exhibit disordered division associated with mitochondrial respiration, anaerobic glycolysis, and enhanced cytomatrix mechanics combined with accelerated biosynthesis. Cytoplasmic fluctuations were shown to be significantly greater in malignant cells than in benign counterparts [12]. Mutations and epigenetic changes that lead to either a loss or gain of function may not immediately impact the acceleration of biochemical processes or protein translation. However, genetic abnormalities that disrupt cellular homeostasis, combined with an excess of ATP produced during aerobic glycolysis, can enhance the micromechanics of the cytomatrix and speed up chemical processes. Due to the rapid acceleration of biosynthetic processes, excessive biomass accumulation from the overproduction of proteins and other substances leads to overcrowding within the cell and an increase in cell size. To maintain cell size, cell survival hinges on its ability to divide and continue its life cycle [30] and triggers malignant cell division.

**Fig. 4:**
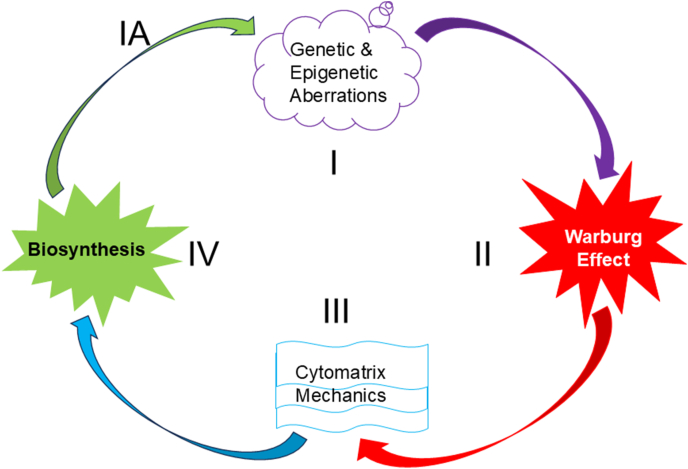
**Triggers of cancer**. The cancer cycle includes genetic alterations (Step I), aerobic glycolysis (Step II), and heightened cytomatrix mechanics (Step III). The latter accelerates biochemical processes (Step IV), leading to biomass accumulation and cell size increases, which trigger cell division. The progression of the cycle can lead to tumor heterogeneity (Step IA). The absence of any cellular steps may inhibit tumor formation.

The current understanding of cytoplasmic consistency, along with the accumulated scientific knowledge, has led to the development of a model of malignant transformation that explains why genetic alterations alone are not enough to trigger tumor formation. Four key factors must coincide to initiate cancer: genetic changes, energy supply, and the mechanics of the cytomatrix that regulate biosynthetic processes (Fig. 4).

The model of tumor development emphasizes the significance of genetic abnormalities, the Warburg effect, intracellular mechanical work, and the rate of chemical reactions, the latter of which is reflected as nucleolar hypertrophy. During the cancer cycle, disorganized synthetic processes, along with chaotic cell division, can result in tumor heterogeneity. The progression of the cancer cycle over time may result in cancer metastasis and drug resistance due to the accumulation of mutations. The focus on the physicochemical aspects of cancer extends the somatic mutation theory of malignant transformation, which has been proven incomplete [31], as it must be coupled with the rate of cell metabolism.

## Funding

This work has been supported, in part, by the Department of Medicine at Baylor College of Medicine. Dr. Tattym Shaiken did not receive any specific grant funding. This work was backed by angel investors, including friends and family who supported Peri-Nuc Labs: Amantai Kurenvekov, Marina Bissekenova, Bakhytzhan Tastulekov, Khadim Urazbayev, Kuanysh Shaiken, and Kairolla Aliakpar.

## Declaration of competing interest

The author declares no conflict of interest.

## Data Availability

Data will be made available on request.
