# Supplementary material for: Cytoplasmic physicochemical factors drive malignant transformation by adapting bioenergetic settings
Source: Biochem Biophys Rep. 2025 Jun 7;43:102079. doi: 10.1016/j.bbrep.2025.102079 (PMC12173752; doi:10.1016/j.bbrep.2025.102079)
Supplement: Multimedia component 4 [file mmc4.docx]

**Table S5. List of actins and actin-binding proteins, and isoforms in the cytomatrix**

| **N** | **Protein and isoform description** | **N** | **Protein and isoform description** |
| --- | --- | --- | --- |
| 1 | actin (alpha, beta, beta-like 2, gamma 1) | 34 | formin-like 2 |
| 2 | actinin (alpha 1, alpha 2, alpha 4) | 35 | gelsolin |
| 3 | actin binding LIM protein 1 | 36 | GIPC PDZ containing family, member 1 |
| 4 | actin-like 6A | 37 | glypican 4 |
| 5 | actin-related protein 2/3 complex (1B, 2, 3, 4, 5, 5 L) | 38 | integrin (alpha 6, beta 3, 4, 6) |
| 6 | anillin actin-binding protein | 39 | inverted formin, FH2 and WH2 domain |
| 7 | annexin (A1, A2, A3, A4, A5, A6, A7, A9, A11, A13) | 40 | LIM domain (7, actin binding 1) |
| 8 | ArfGAP with an SH3 domain, ankyrin repeat and PH domain | 41 | myosin (IB, IC, ID, VB, VI, XIX, heavy chain 9, 10, 14, light chain 6, 12A, 12B) |
| 9 | ARP actin-related protein (1A, 2, 3, 5, 6, 8) | 42 | nebulette |
| 10 | BAI1-associated protein 2 | 43 | parvin, alpha |
| 11 | cadherin (1, 3) | 44 | PDZ and LIM domain (1, 2, 5, 7, 11) |
| 12 | calponin (2, 3) | 45 | plakophilin 4 |
| 13 | capping protein (alpha 1, alpha 2, beta) | 46 | plastin (1, 3) |
| 14 | catenin (alpha 1, alpha 2, beta 1, delta 1) | 47 | pleckstrin domain containing (2, 5, 6, 7, F2, G3, A2, interacting) |
| 15 | claudin (1, 2, 3, 7) | 48 | presenilin associated, rhomboid-like |
| 16 | coactosin-like F-actin-binding protein 1 | 49 | profilin 2 |
| 17 | cofilin 2 | 50 | protocadherin gamma subfamily A, 7 |
| 18 | coronin, actin-binding protein, (1A, 1B, 1C, 7) | 51 | septin (2, 7, 8, 9, 11) |
| 19 | cortactin | 52 | shootin 1 |
| 20 | cysteine-rich PDZ-binding protein | 53 | sorbin and SH3 domain containing 3 |
| 21 | cytoplasmic FMR1-interacting protein 1 | 54 | sorting nexin (1, 2) |
| 22 | cytoskeleton-associated protein (2, 2 L, 5) | 55 | supervillin |
| 23 | desmoplakin | 56 | SWI/SNF related, matrix associated, actin dependent regulator of chromatin |
| 24 | destrin (actin depolymerizing factor) | 57 | syntrophin, (beta 1, beta 2) |
| 25 | drebrin-like | 58 | talin 1 |
| 26 | dynactin (1, 2, 4) | 59 | torsin A interacting protein (1, 2) |
| 27 | dynamin (1, 2) | 60 | tropomodulin 3 (ubiquitous) |
| 28 | fascin actin-bundling protein 1 | 61 | tropomyosin (1, 2, 3, 4) |
| 29 | FAT atypical cadherin 1 | 62 | twinfilin actin binding protein (1, 2) |
| 30 | filamin (alpha, beta) | 63 | villin 1 |
| 31 | flightless I actin-binding protein | 64 | WAS protein family, member 2 |
| 32 | flotillin (1, 2) | 65 | WD repeat domain 1 |
| 33 | formin binding protein 4 |  |  |
